# Discursive constructions of autism on social media: a multilingual analysis across five languages

**DOI:** 10.3389/fpsyg.2026.1769965

**Published:** 2026-04-17

**Authors:** Patricia López-Resa, Ingjerd Skafle, Kevin Rebecchi, Tamara Kalandadze, Anders Nordahl-Hansen, Elia Gabarrón

**Affiliations:** 1 Department of Psychology, Faculty of Health Sciences, University of Castilla-La Mancha, Talavera de la Reina, Spain; 2 Department of Education, ICT and Learning, Østfold University College, Halden, Norway; 3 DIPHE (Development, Individual, Process, Handicap, Education) Research Unit, University Lumière Lyon 2, Lyon, France; 4 Department of Educational Science, University of South-Eastern Norway, Vestfold, Norway

**Keywords:** autism spectrum disorder, cross-language comparison, identity-first language, neurodiversity, person-first language, social media

## Abstract

**Introduction:**

Language choices in autism-related social media discourse reflect ideological models and shape public understanding. Although neuroaffirmative and identity-first language is increasingly visible, cross-linguistic patterns on social media remain poorly understood. These platforms provide a valuable setting to observe how autistic identity and competing medical and neuroaffirmative frameworks circulate internationally.

**Methods:**

This observational multilingual study analyzed 678 public posts collected through stratified keyword sampling in English, Spanish, French, Norwegian, and Georgian. Posts were normalized, anonymized, and classified using multilingual lexicons and sentence-embedding-assisted disambiguation. Variables included theoretical frame (medical, neuroaffirmative, both, neutral), linguistic style (identity-first, person-first), sentiment (−5 to +5), and engagement metrics. Descriptive statistics and longitudinal trend analyses were conducted.

**Results:**

Medical and neuroaffirmative discourse coexisted across languages. Spanish showed the highest medical framing (45.1%), while English (43.4%) and French (36.4%) favored neuroaffirmative discourse; Norwegian and Georgian leaned neutral. Identity-first language predominated overall, with person-first usage most frequent in Norwegian and minimal in Georgian. Sentiment was largely neutral. Engagement varied by language, with identity-first posts generating the highest interaction in English. Neuroaffirmative discourse increased across all languages after 2023.

**Discussion:**

Autism discourse on X/Twitter reflects a global but uneven shift toward neuroaffirmative and identity-first language shaped by linguistic and cultural context, positioning social media as a key arena for renegotiating autistic identity and expertise.

## 1 Introduction

The language used to describe autism is never neutral. Terminological choices carry implicit assumptions about identity, agency, and what is considered conventional, and they shape how autistic people are perceived both socially and institutionally. Across different languages, deficit-oriented accounts that frame autism in terms of pathology or burden coexist with more affirmative discourses that emphasize lived experience, belonging, and identity (Dinishak, 2016). This tension has become particularly visible on social media, where discussions related to language or identity reach broad audiences. Social media have become environments where autistic individuals, families, researchers, organizations and others discuss and exchange contents about autism. These platforms democratize participation, challenge traditional hierarchies, and influence the general public understanding of autism through user-generated narratives.

Several journals and professional bodies have published explicit recommendations regarding the language used to describe autism and/or refer to autistic people (Botha et al., 2023; Bottema-Beutel et al., 2021) and have called for the avoidance of ableist language and for the adoption of terminology that reflect the preferences of autistic communities. For example, the UK-based survey by Keating et al. (2023) informed subsequent editorial policies by showing that identity-first language (“autistic person”) was most widely endorsed among autistic respondents.

Recent studies reinforce this trend. For instance, Taboas et al. (2023) reported that preferences for identity-first vs. person-first language among autism stakeholders in the US aligned broadly with these findings. However, other studies show contrasting preferences. For example, De Laet et al. (2025) found that adults with autism in the Netherlands preferred person-first language and Buijsman et al. (2023) similarly reported a preference for person-first expressions among Dutch adults with autism and their parents. Taken together, these findings suggest that preferences for autism-related terminology are not uniform but may vary systematically across linguistic and cultural contexts. This contrast between predominantly English-speaking and Dutch-speaking settings points to the relevance of language and culture specific norms in shaping how autism is named and understood, an issue that remains underexplored beyond a limited number of languages.

These developments exemplify how models of autism (the medical, the social, and the neurodiversity paradigm) are not only theoretical frameworks but also linguistic regimes that shape what can be said, by whom, and with what authority (Shakes and Cashin, 2020). The medical model conceptualizes autism primarily as an individual disorder requiring diagnosis and treatment, whereas the social model emphasizes the role of social and environmental barriers in producing disability (Shakespeare, 2013). In contrast, the neurodiversity paradigm frames autism as a natural and valuable form of human variation rather than a deficit (Kapp et al., 2013). These frameworks are reflected in recurrent naming practices (e.g., identity-first vs. person-first formulations) and in the use of deficit-oriented vs. affirming descriptors, which are central to how autism is publicly represented (Botha et al., 2023). In this article we have chosen to use identity-first language to align with neuroaffirmative approaches that promote empowerment and reduce stigma.

Previous research has shown that different conceptual models of autism often coexist and interact in complex ways. The medical model, grounded in diagnostic frameworks, positions autism as an individual impairment and therefore favors person-first formulations that linguistically separate the condition from the individual. In contrast, research informed by the social model and, especially, by the neurodiversity paradigm emphasizes autism as a form of human diversity and privileges identity-first language as an affirmation of belonging and collective identity. As Botha et al. (2023) argue, linguistic choice is inseparable from ideological stance: the preference for identity-first language reflects an understanding of autism as an integral part of one's identity rather than a detachable deficit. Similarly, Pellicano and den Houting (2022) highlight that the neurodiversity framework challenges deficit-based assumptions by redefining what counts as “normal” within the autism field. Bottema-Beutel et al. (2021) further argue that avoiding ableist or pathologizing terminology is essential to producing ethically and scientifically rigorous research. Together, these contributions show that language is not a neutral descriptor but a reflection of the conceptual model through which autism is understood—whether as a disorder, difference, or identity, or as a combination of the latter two.

These conceptual distinctions are reflected differently across linguistic and cultural contexts, where discourses of autism are shaped by local expectations, institutional frameworks, and sociocultural norms. In Spanish-speaking contexts, recent research highlights how social and cultural expectations intersect with identity formation. Ramos-Serrano and Garcia-Molina (2025) analyzed narratives of autistic mothers in Spain, showing how stigma and insufficient support networks structure maternal discourse. Similarly, Gargallo-Nieto et al. (2025) documented invisibility, misrecognition, and identity reconfiguration among late-diagnosed women. These studies underscore that, in the Spanish context, discourses on autism remain strongly mediated by gender roles and caregiving expectations, and that identity narratives often emerge in tension with medical and familial perspectives. Similarly, Anglophone research shows that identity-first language (e.g., *autistic person*) is strongly preferred by autistic adults, whereas professionals and family members often use person-first expressions (e.g., *person with autism*) (Keating et al., 2023). Comparable evidence appears in francophone contexts with identity-first terms such as *personne autiste* or simply *autiste* were the most widely accepted (Keating et al., 2023). Within the Norwegian context, there is a notable absence of studies that explicitly examine how autism is named or discursively constructed in public or media discourse. Existing research has primarily focused on diagnostic practices and developmental profiles. For instance, Chahboun et al. (2022) reviewed autism studies and concluded that Norwegian research is shaped simultaneously by international discourses and by local cultural traditions. While informative from a clinical perspective, this line of research does not engage with questions of terminology or public discourse, leaving the linguistic and ideological dimensions through which autism in publicly represented largely unexplored. A convergent pattern is observed in the Georgian context, where no studies were identified that address autism-related terminology or discourse in public communication. Research has remained almost exclusively clinical, focusing on prevalence and linguistic phenotypes (Zirakashvili et al., 2022). Taken together, the Norwegian and Georgian cases illustrate a broader gap in discourse-oriented research, highlighting the relevance of exploring social media as a site where public meanings of autism are negotiated outside clinical frameworks.

At the multilingual level, very limited research has examined autism-related language across languages from a discursive perspective. Existing cross-linguistic studies have primarily addressed terminological preferences or lexical description, rather than broader patterns of language use. For example, research on terminological preferences has documented variability in the use of person-first and identity-first language depending on participants' perspectives and contexts (García-Molina, 2019). Similarly, comparative terminological work has identified systematic differences in autism-related vocabulary across languages, highlighting how naming practices vary across linguistic systems (Luna, 2015). Taken together, these studies illustrate that autism-related language is not uniform across languages, while also indicating that discursive approaches to multilingual autism-related language remain scarce.

Digital environments have proven central to these negotiations of meaning. Early work by Davidson (2008) already identified online spaces as key sites for the emergence of autistic culture and collective identity, showing how digital communication enables the articulation of shared narratives beyond institutional or clinical frameworks. Recent work has shown that social media platforms act not just as information channels, but as arenas where autism-related terminology, identity labels, and ideological positions are actively negotiated (Egner, 2022; Skafle et al., 2024). In these contexts, language choices such as identity-first or person-first formulations operate as discursive strategies through which users manage stigma, claim authority, and position themselves in relation to medical and neuroaffirmative frameworks (Duffin et al., 2025). Importantly, this body of work highlights that autism-related discourse on social media is not static, but evolves over time in response to activist movements, changing platform dynamics, and broader sociocultural shifts (Bakombo et al., 2023). Taken together, these studies position social media as a privileged site for discourse analysis, understood here as a naturalistic communication environment in which meanings circulate publicly and are produced without researcher elicitation, allowing the observation of everyday language practices as they unfold (Schäfer, 2021).

The tension between these frameworks has become especially visible on social media platforms, where individuals themselves increasingly articulate public discourses and self-descriptions that challenge dominant representations through their posts (Akbulut and Bird, 2025; Botha et al., 2022; Skafle et al., 2024). Studying social media is important for understanding how people talk about autism. Platforms like X (formerly Twitter) are key spaces where people share experiences, challenges, stereotypes, and shape autism perception and identity (Skafle et al., 2024). X is still one of the most widely used social media platforms, with almost 600 million monthly users in 2025 (Metricool, 2025). X allows its users to share contents, which makes it possible to study how different views of autism are expressed and discussed across languages and cultures.

The objective of this study is to analyze how autism is discursively constructed in five different languages on X (English, Spanish, French, Norwegian, Georgian), with a particular focus on the use of two contrasting frameworks: the medical model and the neuroaffirmative model. The study aims to examine how these frameworks circulate across linguistic contexts, comparing their prevalence with identity-first vs. person-first language, temporal trends, and engagement, and how online discourse positions autism either as a deficit to be treated or as a valued identity and form of difference.

## 2 Methods

### 2.1 Study design

This observational and descriptive study focuses on two main dimensions: the conceptual models invoked (medical and/or neuroaffirmative) and the linguistic styles employed (identity-first and/or person-first), across five languages: English, French, Spanish, Norwegian, and Georgian. These languages were selected based on the research team's proficiency as well as to ensure representation across different language families and cultural contexts.

### 2.2 Sampling strategy and data collection

We aimed to collect 25 posts per language using seven search-terms selected by the research group as representative of the frameworks under analysis (three terms associated with the neuroaffirmative model; three related to the medical model; and one neutral term), resulting in a total of 175 posts per language (see Table 1). The selection of search terms was guided by a theory-driven approach, aiming to operationalize well-established discursive frameworks in autism research rather than to exhaustively capture all possible community labels or hashtags. Search terms were operationalized as plain-text lexical strings rather than hashtags, selected to ensure functional comparability across languages and to capture autism-related language as it appears in running text.

**Table 1:** Keywords used for data retrieval, grouped by conceptual framework and language.

Term category	English	Spanish	French	Norwegian	Gerogian
Terms associated with the neuroaffirmative model	“Autistic,” “Be autistic,” “proudly autistic”	“Autista,” “ser autista,” “orgullosamente autista”	“Autiste,” “être autiste,” “fier d'être autiste”	“Autistisk,” “være autistisk,” “stolt autist”	“აუტისტი,” “იყოაუტისტი,” “ამაყი აუტისტი”
Terms associated with the medical or deficit model	“With autism,” “suffering autism,” “fighting autism”	“Con autism,” “sufrir autism,” “luchar contra el autismo”	“Avec autism,” “souffrant d'autisme,” “combattre l'autisme.”	“Med autism,” “lidelse autism,” “Bekjempe autisme”	“აუტიზმით,” “აუტიზმით ავად,” “აუტიზმთან ბრძოლა”
Neutral term	“Autism”	“Autismo”	“Autisme”	“Autisme”	“აუტიზმი”

Non-English search terms correspond to literal or functional equivalents of the English expressions listed in the first column.

The objective of this sampling strategy was not to approximate the full volume of autism-related content on X, but to construct a controlled, cross-linguistically comparable corpus that would allow fine-grained discursive analysis across predefined conceptual strata. In contrast to large-scale sentiment studies based on millions of posts, which prioritize breadth and predictive modeling, the present design follows a stratified, theory-driven approach oriented toward interpretative comparison of discursive patterns. The unit of analysis is therefore discursive configuration rather than platform-wide prevalence (Jungherr, 2018).

Search terms were further selected based on their lexical compositionality and cross-linguistic translatability, that is, their ability to be expressed as semantically equivalent multiword expressions combining autism (or its equivalents) with evaluative or relational predicates across all five languages. This sample size was chosen for both pragmatic and statistical reasons. Quantitatively, 175 observations per language balance precision and feasibility, yielding an approximate 7–7.5% margin of error at a 95% confidence level, sufficient to detect cross-linguistic differences in discursive frames. Importantly, these calculations refer to comparisons within the defined sampling frame and should not be interpreted as estimates of overall platform-level distributions.

Crucially, the allocation of 25 posts to each keyword follows the logic of optimal (Neyman) allocation in stratified designs, where the per-stratum sample *n*_h_ is proportional to the stratum weight and its within-stratum variability. Pilot retrievals showed similar within-term variability and equal weighting across strata, supporting equal allocation. From a qualitative standpoint, this per-term size provided sufficient lexical and thematic breadth, keeping manual coding and cross-language validation tractable. This balanced stratification ensures that each conceptual frame is analytically visible across languages, avoiding dominance by high-frequency but conceptually narrow terms. Overall, the stratified, equal-allocation design combines methodological robustness with practical manageability (Jerzy Neyman, 1934; Kish, 1965; Cochran, 1977). The selected keywords used for data retrieval are presented in Table 1.

Data collection was conducted by assigning each language to a researcher with native or daily-use proficiency in that language. Searches in Spanish, Norwegian, French, and Georgian were conducted by researchers who were native speakers of those languages. Searches in English were conducted by researchers already involved in the project who regularly use English in their academic and professional practice. Searches were conducted on X using the “advanced search” function for each selected term. For every keyword, the last 25 posts (counting backward from September 17, 2025) were manually extracted, including both the text of the post and its associated metadata. In cases where 25 posts were not available for a given keyword, the maximum number of available posts was collected. As a result, the temporal range of the dataset varies across search terms and languages, and in some cases extends back to earlier years when recent activity was limited. Because retrieval was bounded by the most recent available posts per term, the resulting corpus represents a structured cross-sectional snapshot with retrospective temporal depth, rather than a comprehensive longitudinal archive of platform activity. Across all languages and search terms, the retrieved posts span from 2011 to September 17, 2025, depending on the availability of recent content for each keyword.

To minimize algorithmic variability, all searches were conducted within the same predefined time window, using the same search interface (“advanced search”) and identical query parameters across languages. While user-level algorithmic differences cannot be fully eliminated on X, conducting searches by linguistically competent researchers was prioritized to ensure accurate identification and interpretation of relevant content in each language. This approach reflects a trade-off between algorithmic consistency and linguistic validity, as discussed in prior work on social media data collection (Olteanu et al., 2019).

The collection window was documented with dates, times, time zone, and the same API used, thereby ensuring transparency and replicability. Posts were included in the dataset if they (a) were publicly accessible at the time of collection, (b) contained one of the predefined search terms in the body of the text, and (c) were written in one of the five target languages (English, French, Spanish, Norwegian, or Georgian). Exclusion criteria were applied systematically prior to coding. Posts were excluded if they (a) constituted automated or bot-generated content, (b) contained promotional or spam material unrelated to autism discourse, (c) consisted solely of hyperlinks without contextual text, or (d) were written in languages other than the five under analysis. Automated messages were defined as posts generated by accounts exhibiting clear signs of automation, such as repetitive or templated content and the absence of interactional features. Spam was defined as posts containing promotional or irrelevant content unrelated to autism discourse, including messages consisting solely of external links without accompanying contextual text.

All retrieved posts, whether an original post, a reply, or a quote-post, was treated as an autonomous communicative units (Krippendorff, 2018). Forwarded posts without additional commentary were excluded, as they do not add original discursive content.

Metadata (date, engagement metrics and language) were retained for all included posts to enable descriptive comparison across languages and discursive categories. Post was stored together with the search term that triggered its retrieval, making it possible to trace and evaluate potential biases introduced by keyword selection, as recommended by Krippendorff (2018) and MacQueen et al. (1998). This design acknowledges methodological concerns raised in the literature: hashtags and keywords are used unevenly across communities (Tufekci, 2014), the Twitter public API may underrepresent certain content compared to the full stream (Morstatter et al., 2021), and biases can emerge at different stages of the research cycle (Olteanu et al., 2019). Accordingly, identity-based hashtags (e.g., *#ActuallyAutistic*) were excluded, as they function as non-compositional, language-specific indexical markers and do not have functionally equivalent lexical realizations across the languages examined. To address these risks, the sampling strategy combined targeted terms from both discursive frames with a more general keyword (“autism” and equivalents), following the methodological recommendations of Olteanu et al. (2019).

Taken together, the dataset should be understood as an analytically constructed, multilingual corpus designed to enable systematic comparison of discursive frames across languages, rather than as a statistically exhaustive or fully representative sample of all autism-related communication on X.

### 2.3 Coding

In terms of theoretical models, entire posts wew coded at the message level as “Medical,” “Neuroaffirmative,” “Both,” or “Neutral.”

Operationally, classification into “Medical” or “Neuroaffirmative” was based on the presence of lexical items or multiword expressions associated with each conceptual framework, as defined in the multilingual lexicon described below (see Section 2.4). Posts were coded as “Medical” when they framed autism primarily in terms of disorder, deficit, suffering, pathology, treatment, or burden (e.g., references to diagnosis, impairment, struggle, or fighting autism). Posts were coded as “Neuroaffirmative” when they framed autism as identity, difference, pride, diversity, or belonging (e.g., references to being autistic, autistic identity, neurodiversity, pride, or community). Classification did not depend solely on explicit first-person self-identification (e.g., “I am autistic”), but on the semantic framing of autism within the post.

Lexical matching was applied to normalized text (after removal of URLs and formatting elements), and orthographic or minor stylistic variations, including capitalization and embedded hashtags (e.g., “#autistic”), were treated equivalently when the lexical root corresponded to items in the predefined lexicon. Posts containing lexical triggers from both conceptual repertoires were coded as “Both.” Posts lacking any lexicon-based indicators of either framework were coded as “Neutral.”

Regarding linguistic styles, entire posts were likewise coded at the message level as “Identity-first,” “Person-first,” “Both,” or “None.”

Identity-first language was operationalized as constructions in which autism functions as an attributive or nominal identity label (e.g., “autistic person,” “autistic adult,” or language-specific equivalents), whereas person-first language was defined as constructions that grammatically separate the person from the condition (e.g., “person with autism,” “child with autism,” or equivalents). These categories were identified using language-specific regular expressions capturing canonical syntactic patterns in each language. Posts were coded as “Both” when both constructions appeared within the same message. Posts that referred to autism without employing either structure (e.g., generic references such as “autism is…”) were coded as “None.”

### 2.4 Data analysis

All data processing was performed in Python. Texts were normalized (Unicode NFC, Normalization Form Canonical Composition), lowercased, and cleaned of URLs, mentions, and hashtags. Language identification was conducted using *langdetect*, supplemented by script-based heuristics for Georgian to reduce misclassification due to its distinct alphabet.

To support post-level classification, a multilingual conceptual lexicon was constructed in five languages (English, Spanish, French, Norwegian, and Georgian). The lexicon was developed deductively, drawing on academic literature and autism-advocacy sources, and was designed to operationalise the two conceptual frameworks under analysis (medical vs. neuroaffirmative). For each language, the lexicon included lexical items and multiword expressions associated with identity, pride, and self-affirmation, as well as expressions framing autism in terms of illness, suffering, or struggle. Morphological variants and idiomatic expressions were included to ensure coverage beyond the initial sampling seeds.

Although seed terms guided initial data retrieval, post-level classification was carried out independently using a rule-based system supported by a multilingual lexicon developed from academic literature and autism-advocacy sources (Jardim et al., 2021). Rule-based classification relied on surface-level lexical matching: the presence of any lexicon item triggered assignment to the corresponding conceptual model. Posts containing terms from both frameworks were labeled as “Both,” while posts without any matching items were labeled as “None.”

Posts were classified along two dimensions: conceptual model (medical vs. neuroaffirmative) and linguistic style (identity-first vs. person-first). Representative forms included “autistic person” vs. “person with autism” in English, with direct analogs in Spanish, French, and Norwegian. For Georgian, verbal constructions expressing “has autism” were treated as person-first to reflect language-internal structures. Identity-first and person-first language was identified using language-specific regular expressions capturing canonical constructions in each language, applied uniformly across languages using functionally equivalent patterns.

For Georgian cases with ambiguous rule-based assignment, cross-lingual sentence embeddings (LaBSE, Language—Agnostic BERT Sentence Embedding; Feng et al., 2022) were used to compute cosine similarity between each post and prototypical phrases, following established procedures for low-resource languages. Similarity thresholds were calibrated empirically within the corpus.

Descriptive linguistic metrics were calculated, including token counts, mean tokens per post, type-token ratio, and mean word length. Semantic co-occurrence networks were constructed using NetworkX to identify thematic associations across languages. Visualizations (e.g., heatmaps, word clouds) were used as comparative tools to display these descriptive measures across languages and discursive categories, facilitating the identification of cross-linguistic patterns rather than constituting an additional analytical layer.

Sentiment analysis was lexicon-based and computed over the full normalized textual content of each post. For English, the VADER sentiment lexicon was used, given its suitability for social media text. For Spanish, French, and Norwegian, language-specific polarity lexicons were applied, adapted to each language and used separately to avoid cross-linguistic contamination. For Georgian, a sentiment lexicon was created through translation and manually validated via concordance checks to ensure contextual appropriateness. Polarity scores ranged from −5 (strongly negative) to +5 (strongly positive), representing the overall affective orientation of each post as a communicative unit.

Given typological variation among the five languages—including analytic morphology (English, Norwegian), fusional morphology (Spanish, French), and polysynthetic verbal morphology (Georgian)—metrics were interpreted within linguistic context rather than used for direct structural comparison.

Interaction metrics (replies, reposts, likes, and views) were analyzed to assess user engagement. Each post's engagement profile was linked to its discourse model (medical, neuroaffirmative, neutral, both) and linguistic style (identity-first, person-first, both, none). Mean interaction values were calculated for each category and language, and comparative distributions were visualized. These metrics were included to provide contextual information about the relative visibility and circulation of different discursive patterns on the platform, rather than to test causal or inferential claims. Because social media engagement data are typically skewed, results were summarized descriptively and inspected for outliers; inferential statistics were not applied, given variability in posting volume and audience size across languages.

### 2.5 Ethical considerations

The study was evaluate by the Norwegian Agency for Shared Services in Education and Research (SIKT), ensuring compliance with ethical standards for privacy, data protection, and responsible handling of social media data (Ref: 562563).

## 3 Results

### 3.1 Corpus and classification overview

A total of 678 posts were collected across all languages: 175 in English, 175 in Spanish, 173 in French, 80 in Norwegian, and 75 in Georgian (Table 2).

**Table 2:** Cross-linguistic distribution of medical, neuroaffirmative, and neutral discourses.

Category	English (*n* = 175)	Spanish (*n* = 175)	French (*n* = 173)	Norwegian (*n* = 80)	Georgian (*n* = 75)
Discourse models					
Medical	39.4% (*N* = 69)	45.1% (*N* = 79)	31.8% (*N* = 55)	31.2% (*N* = 25)	37.3% (*N* = 28)
Neuroaffirmative	43.4% (*N* = 76)	38.3% (*N* = 67)	36.4% (*N* = 63)	35.0% (*N* = 28)	28.0% (*N* = 21)
Both	6.3% (*N* = 11)	1.1% (*N* = 2)	9.2% (*N* = 16)	0.0% (*N* = 0)	12.0% (*N* = 9)
Neutral	10.9% (*N* = 19)	15.4% (*N* = 27)	22.5% (*N* = 39)	33.8% (*N* = 27)	22.7% (*N* = 17)
Language style					
Person-first	21.7% (*N* = 38)	17.1% (*N* = 30)	26.6% (*N* = 46)	28.7% (*N* = 23)	1.3% (*N* = 1)
Identity-first	42.3% (*N* = 74)	38.3% (*N* = 67)	39.9% (*N* = 69)	35.0% (*N* = 28)	32.0% (*N* = 24)
Both	3.4% (*N* = 6)	1.1% (*N* = 2)	1.7% (*N* = 3)	0.0% (*N* = 0)	0.0% (*N* = 0)
None	32.6% (*N* = 57)	43.4% (*N* = 76)	31.8% (*N* = 55)	36.2% (*N* = 29)	66.7% (*N* = 50)

Regarding the theoretical models, the results show that Spanish posts are dominated by the Medical model, whereas English posts display a more balanced distribution between Medical and Neuroaffirmative frames. French posts also lean toward these two categories, with a slightly stronger presence of Neuroaffirmative content, while Georgian posts stand out for the predominance of Neutral. Norwegian posts are more evenly distributed across Medical, Neuroaffirmative, and Neutral.

A descriptive inspection of high-frequency lexical items (see Supplementary material) indicates that, beyond the core autism-related terms common across languages, evaluative and action-oriented vocabulary varies systematically by discourse model. In the Medical model, posts frequently include clinical and struggle-related lexicon such as sufrir, luchar, contra, fight, and comparable verbs across languages, framing autism in terms of disorder, hardship, or impairment. In contrast, the Neuroaffirmative model is characterized by the prominence of identity labels—autistic, autista, autiste, autistisk, აუტისტი—together with pride markers such as proudly and orgullosamente, constructing autism as identity and belonging. Posts coded as both show the co-occurrence of clinical and identity-oriented terms within the same message, often accompanied by references to support or inclusion. Neutral posts tend to foreground general references to autism (autism, autismo, autisme, autisme in Norwegian, Norwegian, აუტიზმი) alongside high-frequency verbs such as have, avoir, jeg, or language-specific equivalents, pointing to a more descriptive and informational register oriented toward explanation or knowledge-sharing.

On the linguistic styles, the analysis reveals that Identity-first is consistently the most frequent choice across languages, particularly in Spanish and French, while Person-first is relatively stronger in Norwegian. Georgian posts, in contrast, are most often categorized as None, showing the absence of explicit reference to either style.

A parallel inspection of high-frequency lexical patterns (see Supplementary material) shows that Identity-first posts are characterized by the recurrent presence of identity labels such as autistic, autista, autiste, autistisk, and აუტისტი, typically embedded in self-referential or collective formulations. Person-first posts display constructions such as with autism, con autismo, avec autisme, med autisme, and აუტიზმით, which grammatically foreground the person in relation to autism and often co-occur with nouns referring to everyday life or care contexts. Posts categorized as both contain both constructions within the same message, whereas none posts refer to autism without employing either identity-first or person-first syntactic structures, instead relying on more general or descriptive formulations.

### 3.2 Semantic networks

Across languages, the semantic network (see Supplementary material) are centered on the main autism-related lexical forms, but the structure of each network is defined by the sets of verbs, evaluative terms, and relational expressions that cluster around these central nodes and vary by discourse model. In the Medical model, possessive verbs such as *tener* (Spanish) and *have* (English), together with deficit-oriented terms like *disorder, diagnóstico*, and *tratamiento*, frame autism as something to be possessed, endured, or treated. The Neuroaffirmative model is structured around existential verbs such as *ser* (Spanish) and ê*tre* (French), supported by identity labels (*autistic, autista, autiste*) and affirming expressions such as *proudly, orgullosamente, inclusion, diversity*, and *community*. French displays a hybrid structure where definitional formulations (ê*tre autiste*) coexist with affective ones (*souffrir, mon enfant*), while Norwegian and Georgian emphasize support and awareness (*støtte*-suppor-, *inkludering*-inclusion-, მხარდაჭერა-support-, ოჯახი-family-, together with global hashtags such as *#worldautismday* and *#autismawareness*), Both Norwegian and Georgian networks are therefore more closely aligned with the Neutral category.

In the language network, the central nodes correspond to the different forms of “autism” in each language (*autism, autisme, autismo*, აუტიზმი), around which the linguistic styles are organized. Identity-first expressions such as *autistic* (English), *autista* (Spanish), *autiste* (French), *autistisk* (Norwegian), and აუტისტი (Georgian) form a dense cluster, often linked to terms of pride or belonging such as *proudly, orgullosamente, neurodivergent*, and *neurodiversité*. Person-first constructions, including *with autism, con autismo, avec autisme, barn med autisme* (Norwegian), and აუტიზმით (Georgian), establish a parallel cluster where the condition is subordinated to the person. The *Both* category emerges when these two types of expressions co-occur in the same message, while *None* appears in more general formulations such as *autism is* (English), *el autismo es* (Spanish), or *l'autisme est* (French), which describe autism without adopting either linguistic style. This organization shows a greater presence of Identity-first in English, Spanish, and French, stronger Person-first usage in Norwegian, and a relatively higher proportion of None in Georgian.

Taken together, the two networks demonstrate that the analytical categories emerge directly from the observed lexical patterns: identity-first and the Neuroaffirmative model are reinforced through identity labels, existential verbs, and affirming vocabulary; person-first and the Medical model are supported by person-centered constructions, possessive verbs, and lexicon of suffering; both emerges when repertoires overlap within the same cluster; and neutral appears through the prevalence of support, inclusion, and awareness discourse, particularly prominent in Norwegian and Georgian.

### 3.3 Sentiment analysis

**Figure 1:**
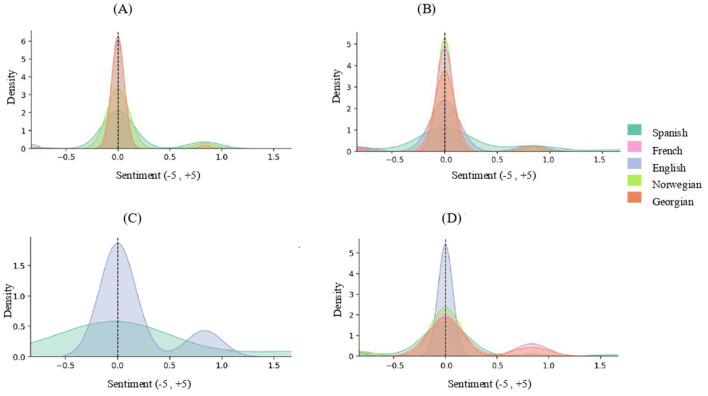
Cross-linguistic distribution of sentiment scores across theoretical models. **(A)** Medical, **(B)** Neuroaffirmative, **(C)** Both and **(D)** Neutral discourses. This figure shows kernel density distributions of sentiment scores (−5 to +5) for posts classified under four theoretical models—**(A)** Medical, **(B)** Neuroaffirmative, **(C)** Both, and **(D)** Neutral—with colored curves representing different languages. Across models, sentiment scores are predominantly concentrated around neutral values (near 0), although the degree of dispersion varies by model and language. The Medical and Neuroaffirmative panels show tightly clustered, sharply peaked distributions across most languages, indicating low variability in sentiment, whereas the Both model displays broader and more heterogeneous distributions, with wider spreads toward both negative and positive values for some languages. The Neutral model again shows strong clustering around neutrality, with minor asymmetries or secondary peaks in some languages. Overlapping curves indicate similar sentiment distributions across languages within each model, while differences in peak shape and spread reflect cross-linguistic variation in sentiment intensity associated with each theoretical framework.

The distribution of sentiment by theoretical models (Figure 1) shows that the *Both* model registers the highest means across the dataset, with English reaching the maximum positive value (*M* = 0.30, SD = 0.89) and French also showing a positive tendency (*M* = 0.16, SD = 0.33), while Spanish remains neutral (*M* = 0.00, SD = 0.00). The *Medical* model is consistently close to neutrality in all languages, with slightly positive means in English (*M* = 0.07, SD = 0.39) and Norwegian (*M* = 0.07, SD = 0.31), a slightly negative mean in French (*M* = −0.03, SD = 0.28), and complete neutrality in Georgian (*M* = 0.00, SD = 0.00). The *Neuroaffirmative* model shows a more positive profile in English (*M* = 0.16, SD = 0.43), while Spanish, French, and Norwegian remain near zero (0.01–0.03, SD = 0.16–0.26), and Georgian shows a slightly negative value (*M* = −0.03, SD = 0.18). The *Neutral* model yields mixed results: English is exactly neutral (*M* = 0.00, SD = 0.28), Spanish (*M* = 0.16, SD = 0.30) and Georgian (*M* = 0.19, SD = 0.34) display positive means, while French (*M* = −0.03, SD = 0.19) and Norwegian (*M* = −0.04, SD = 0.22) register negative values.

**Figure 2:**
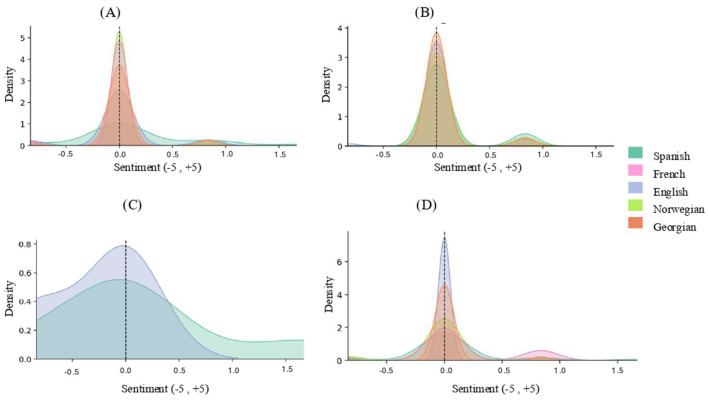
Cross-linguistic distribution of sentiment scores across linguistic styles. **(A)** Identity-first, **(B)** Person-first, **(C)** Both and **(D)** Neutral styles. This figure presents kernel density distributions of sentiment scores (−5 to +5) across linguistic styles—**(A)** Identity-first, **(B)** Person-first, **(C)** Both, and **(D)** Neutral—with colored curves representing different languages. In the Identity-first and Person-first panels, sentiment distributions across languages are tightly clustered around neutral values, showing sharp central peaks and limited dispersion, which indicates relatively homogeneous sentiment patterns across languages for these styles. In contrast, the Both style exhibits broader and more diffuse distributions, with greater spread toward both negative and positive sentiment values and clearer cross-linguistic differences in curve shape and width. The Neutral style again shows a strong concentration around neutrality, although some languages display secondary peaks or slight rightward skew, suggesting modest variation in positive sentiment. Overlap between curves reflects similarity in sentiment distributions across languages within each linguistic style, whereas differences in dispersion and peak shape indicate cross-linguistic variability in how sentiment is expressed across styles.

Turning to the distribution by linguistic styles (Figure 2), the overall pattern remains concentrated around neutrality, though with some notable differences. In the *Identity-first* style, English reaches the highest positive mean (*M* = 0.18, SD = 0.67), Georgian shows a slightly negative tendency (*M* = −0.03, SD = 0.19), and Spanish, French, and Norwegian remain close to neutrality (0.01–0.03, SD = 0.25–0.29). In cases without a defined style (*None*), Georgian records the most positive mean (*M* = 0.19, SD = 0.35), while French (*M* = −0.02, SD = 0.24) and Norwegian (*M* = −0.03, SD = 0.23) lean slightly negative; English (*M* = 0.04, SD = 0.28) and Spanish (*M* = 0.03, SD = 0.19) remain near zero. The *Person-first* style is predominantly positive, with higher values in English (*M* = 0.11, SD = 0.45) and Norwegian (*M* = 0.07, SD = 0.27), followed by Spanish (*M* = 0.06, SD = 0.22) and French (*M* = 0.04, SD = 0.20), while Georgian remains strictly neutral (*M* = 0.00, SD = 0.00). Finally, the *Both* category shows greater variability: English remains positive (*M* = 0.14, SD = 0.75), Spanish neutral (*M* = 0.00, SD = 0.00), and French negative (*M* = −0.28, SD = 0.39).

Overall, sentiment distributions cluster around neutrality, though some categories and languages deviate in significant ways. The most positive values appear in English under the *Both* model (*M* = 0.30, SD = 0.89) and in Georgian under the *Neutral* style (*M* = 0.19, SD = 0.35). By contrast, the most negative values are concentrated in French—particularly in the *Both* style—and, to a lesser extent, in Norwegian.

### 3.4 Temporal evolution of discourses on autism

In total, the corpus includes 678 posts produced by 602 unique users. The distribution by language is as follows: English (167 users, 175 posts), Spanish (172 users, 175 posts), French (157 users, 173 posts), Norwegian (65 users, 79 posts), and Georgian (41 users, 74 posts). This reflects unequal language presence and participation density across communities.

The temporal analysis describes how discourses on autism are distributed over time within the retrospectively constructed corpus, rather than constituting a prospective longitudinal design. Because posts were collected by retrieving the most recent available messages for each search term and language, earlier years are represented when recent activity was limited, resulting in a dataset spanning from 2011 to 2025. Within this corpus-based temporal distribution, discourses on autism show a clear transformation over time, both in terms of theoretical models and linguistic styles.

**Figure 3:**
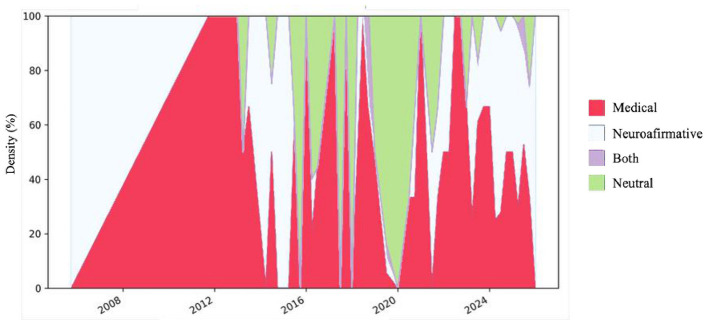
Longitudinal evolution of theoretical discourse models across languages. Colored areas represent the relative proportion (%) of Medical, Neuroaffirmative, Both, and Neutral discourses over time.

Regarding the models (Figure 3), the analysis shows a global trend marked by a shift from the historical predominance of the medical framework toward greater discursive plurality, with the neuroaffirmative model gaining sustained ground in recent years. In the English-language dataset, after clear medical dominance in 2011, the neuroaffirmative approach gained visibility between 2014 and 2024, leading to a more diverse panorama in 2025 in which all four frameworks coexisted. In the Spanish corpus, the medical model remained dominant from 2011 to 2022, but from 2023 onwards it lost ground in favor of the neuroaffirmative perspective and, to a lesser extent, neutral positions. In the French dataset, fluctuations were more pronounced, with alternating predominance of the four models across years and a more balanced distribution emerging in 2025. In Georgian, medical and neutral perspectives prevailed between 2015 and 2020, while the neuroaffirmative discourse became clearly consolidated from 2023 onwards. In Norwegian, the discourse was initially neutral in 2013, shifted entirely toward the neuroaffirmative in 2023, and diversified again in 2025 with the presence of multiple frameworks. Overall, the results indicate an erosion of the medical model's hegemony and the rise of the neuroaffirmative perspective as the most expanding frame, accompanied by the emergence of a plural discursive space where mixed and neutral approaches also gain relevance.

**Figure 4:**
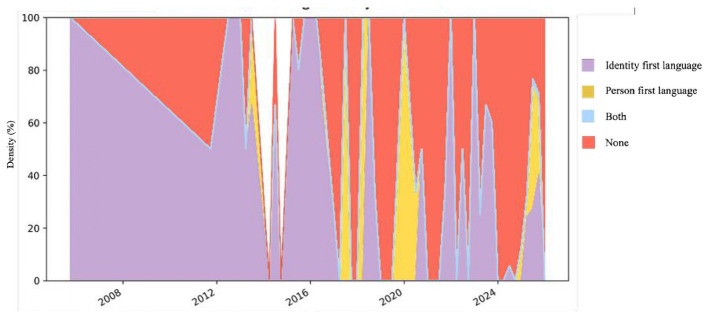
Longitudinal evolution of linguistic styles across languages. Colored areas represent the relative proportion (%) of identity-first, person-first, both, and neutral styles over time.

With regard to linguistic styles (Figure 4), the evolution follows a parallel trend. Identity-first formulations have progressively gained prominence, although they coexist with person-first expressions, mixed uses of both styles, and utterances without explicit reference. In English, identity-first remained stable and consolidated as the predominant style in the last decade, though in 2025 it coexisted with person-first and mixed formulations. In Spanish, person-first was dominant until 2022, but in recent years identity-first and mixed expressions increased, reflecting a diversification of styles. French shows fluctuations throughout the period, with a clearer balance in 2025 between identity-first, person-first, and mixed formulations. In Georgian, the absence of explicit references (*none*) predominated in earlier years, while more recently identity-first gained prominence. In Norwegian, early uses were scattered and heterogeneous, but from 2023 onwards identity-first became clearly predominant, accompanied by some mixed uses. Overall, the evolution of linguistic styles confirms the growing visibility of identity-first, in parallel with the diversification observed in theoretical models.

### 3.5 Interaction metrics

The analysis of the interaction metrics shows systematic differences in interaction levels across discourse models and linguistic styles, with notable variations across languages (Table 3).

**Table 3:** Mean interaction metrics by discourse model and language.

Model/style	Language	Replies	Reposts	Likes	Views
Discourse models					
Medical model	EN	1.80 (11.52)	2.23 (11.59)	22.43 (129.82)	809.72 (4499.09)
	SP	0.38 (0.57)	0.50 (1.90)	2.35 (4.54)	121.88 (280.96)
	FR	2.43 (3.52)	55.86 (138.76)	111.29 (231.57)	2478.93 (3990.22)
	GE	0.12 (0.47)	0.00 (0.00)	0.79 (2.07)	21.31 (69.28)
	NO	1.57 (4.13)	1.81 (5.45)	16.27 (35.82)	710.14 (1600.27)
Neuroaffirmative model	EN	1.24 (3.29)	2.82 (13.76)	16.71 (76.71)	289.84 (453.28)
	SP	3.80 (16.97)	16.42 (74.96)	44.68 (177.93)	792.73 (2646.90)
	FR	1.29 (3.82)	15.86 (63.66)	82.95 (411.36)	4756.43 (31514.76)
	GE	0.00 (0.00)	0.00 (0.00)	0.33 (0.58)	10.67 (16.77)
	NO	0.57 (1.87)	0.00 (0.00)	5.93 (21.90)	424.07 (1014.42)
Both	EN	5.22 (20.54)	11.87 (48.85)	130.73 (508.22)	4012.79 (18160.06)
	SP	0.88 (1.75)	1.80 (4.40)	7.17 (13.64)	769.02 (3064.87)
	FR	0.50 (0.71)	0.00 (0.00)	1.00 (0.00)	30.50 (28.99)
	GE	1.46 (3.62)	0.39 (1.10)	5.89 (9.83)	513.11 (962.12)
	NO	0.40 (0.59)	0.12 (0.47)	1.66 (5.18)	100.19 (295.61)
Neutral	EN	3.33 (5.83)	5.11 (14.96)	37.00 (90.27)	1601.67 (3926.79)
	SP	0.50 (0.71)	4.00 (5.66)	1.50 (2.12)	22.00 (128.69)
	FR	0.21 (0.43)	1.00 (1.52)	3.57 (5.26)	167.29 (192.15)
Language style					
Identity-first	EN	4.15 (18.77)	6.58 (23.28)	91.55 (399.01)	3453.76 (17740.20)
	SP	0.95 (4.81)	0.57 (3.91)	9.55 (69.40)	99.27 (293.68)
	FR	0.84 (1.72)	1.56 (4.01)	6.69 (13.26)	732.16 (2998.53)
	GE	0.00 (0.00)	0.00 (0.00)	0.04 (0.21)	2.17 (6.19)
	NO	1.90 (4.40)	1.23 (4.80)	9.93 (22.72)	663.27 (1250.70)
Person-first	EN	3.17 (15.09)	8.36 (47.91)	68.98 (372.58)	1514.64 (6847.71)
	SP	3.41 (16.70)	16.69 (76.47)	37.97 (168.31)	805.88 (2692.09)
	FR	2.26 (4.15)	43.26 (114.95)	178.39 (556.63)	8854.65 (42253.70)
	GE	0.22 (0.61)	0.00 (0.00)	1.15 (2.38)	29.54 (80.59)
	NO	1.27 (3.51)	1.24 (3.80)	14.21 (33.06)	622.36 (1498.45)
Both	EN	1.43 (3.72)	3.50 (14.30)	21.02 (81.28)	549.52 (1818.42)
	SP	0.83 (3.07)	7.49 (40.14)	15.57 (76.72)	613.74 (1961.25)
	FR	0.00 (0.00)	0.00 (0.00)	1.00 (2.29)	31.22 (79.64)
	GE	0.50 (1.75)	0.00 (0.00)	5.19 (20.48)	383.94 (950.73)
	NO	0.27 (0.45)	0.65 (1.92)	2.00 (4.06)	143.80 (247.84)
None	EN	2.17 (4.83)	0.50 (0.84)	9.67 (19.33)	364.67 (560.30)
	FR	0.00 (0.00)	0.67 (0.58)	0.67 (0.58)	88.67 (85.49)
	SP	0.00 (0.00)	0.00 (0.00)	2.00 (0.00)	119.00 (128.69)

Values represent mean interaction metrics, with standard deviations in parentheses. Language codes are as follows: EN, English; SP, Spanish; FR, French; GE, Georgian; NO, Norwegian.

Overall, the results indicate that higher interaction levels are observed for posts aligned with the Medical model in French and Spanish, whereas the Neutral model is associated with higher interaction values in English. With regard to linguistic styles, the None category shows higher interaction levels in French and Spanish, while in English Identity-first language is associated with higher interaction. Norwegian and Georgian, by contrast, show generally low interaction levels across models and styles, with no consistent pattern, except for a slight advantage of Identity-first language in Norwegian.

## 4 Discussion

The present study examined how autism is discursively constructed across five languages on X/twitter within a stratified multilingual corpus, revealing that linguistic choices about autism are not merely stylistic but reflect broader ideological and cultural orientations. Across the dataset, medicalized and deficit-oriented framings continue to coexist alongside neuroaffirmative narratives that emphasize identity, belonging, and community. This duality is consistent with recent findings suggesting that public discussions of autism may be undergoing a gradual paradigmatic shift from pathology to neurodiversity (Bottini et al., 2024). The coexistence of these frameworks on social media mirrors the broader transformation of autism research and public communication observed over the past decade, as identity-first language and lived-experience accounts have increasingly challenged professional medical dominance (Zajic and Gudknecht, 2024). Beyond terminology, these discursive choices may have implications for wellbeing. Research on autistic adults has shown that experiences of stigma and misunderstanding are strongly associated with poorer mental health outcomes, including higher levels of anxiety and depression (Leadbitter et al., 2021; Cage et al., 2018). In this context, identity-affirming language and community-led narratives can function as resources for self-understanding and social validation. Online environments where autistic individuals articulate identity-based perspectives may therefore contribute to forms of peer support and collective meaning-making that are relevant for psychological wellbeing (Gillespie-Lynch et al., 2014).

Our cross-linguistic analysis revealed both convergence and divergence among languages. English and French corpora exhibited the highest frequency of neuroaffirmative discourse, while Spanish remained predominantly medicalized and Georgian and Norwegian showed more neutral or descriptive tendencies. These patterns reflect differences in the maturity of autistic self-advocacy movements, media ecosystems, and institutional policies. Studies of English-language digital spaces have shown that autistic people increasingly use social media to reclaim narrative authority and reframe autism through identity and pride (Botha and Cage, 2022; Egner, 2022) comparable processes have been reported in French, where *personne autiste* has become the preferred form among self-advocates (Geelhand et al., 2023) and in Spanish-speaking contexts where activist accounts and mothers' networks are beginning to challenge clinical representations (Ijalba, 2016; Mendiguren Galdospin et al., 2023). By contrast, in Norway and Georgia, public discourse remains closer to awareness and support frameworks rather than explicit (Bakka and Grue, 2025; Solvang, 2000). In a recent Norwegian study by Skafle et al. (2024), the autistic participants were negative toward the deficit/impairment-oriented focus in the Norwegian public health sources concerning autism. According to the participants, they have to search on social media to find more neuro-affirmative information regarding different topics on autism. This reinforces our finding that public discourse in Norway remains predominantly medicalized, prompting autistic individuals to seek neuroaffirmative content elsewhere.

Within this broader cross-linguistic pattern, differences in linguistic framing further emerge in the contrast between identity-first and person-first language. The predominance of identity-first language in English, French, and Spanish parallels empirical survey data. Large-scale studies confirm that autistic adults overwhelmingly prefer identity-first expressions such as *autistic person* over person-first alternatives, arguing that these formulations acknowledge autism as integral to identity (Bury et al., 2023; Lai et al., 2023). By contrast, professionals and caregivers still favor person-first terminology, which many autistic respondents experience as distancing or pathologizing. This tension is also visible in research publishing: Zajic and Gudknecht (2024) analyzed over 12,000 abstracts from autism journals and found that person-first language remains dominant in academia despite its declining popularity among autistic communities. The persistence of these stylistic divides within our corpus therefore reflects a broader lag between community and institutional discourse.

These discursive differences are also relevant when considered through the lens of psychological wellbeing. Research with autistic adults consistently shows that experiences of stigma, misunderstanding, and deficit-based representations are associated with poorer mental health outcomes, including elevated anxiety, depression, and reduced quality of life (Cage et al., 2018; Leadbitter et al., 2021). From this perspective, linguistic framings that emphasize pathology or burden may contribute to social environments in which autistic identities are implicitly devalued. Conversely, identity-affirming narratives—such as those emerging in neurodiversity-oriented communities—can provide interpretative frameworks that support self-acceptance, collective identity, and a sense of belonging. Social media platforms play a particularly important role in this process. Studies of autistic participation in online communities show that digital spaces often function as sites of peer support, knowledge exchange, and identity development, allowing autistic individuals to share lived experiences and challenge dominant medical narratives (Gillespie-Lynch et al., 2014; Botha and Cage, 2022). The multilingual discursive patterns identified in our corpus therefore have potential implications beyond language itself: they shape the kinds of narratives that circulate within different linguistic publics and may influence how autism is understood by autistic individuals, families, and the broader society. In this sense, discursive environments on social media can indirectly contribute to wellbeing by facilitating—or constraining—access to validating representations and supportive communities.

In this study, we found that the distribution of posts in English was nearly equal between the medical and neuroaffirmative approaches (39.4% vs. 43.4%). However, there was a clear dominance of identity-first language compared to person-first language (42.3% vs. 21.7%). These findings are consistent with previous research indicating that identity-first language is strongly preferred by autistic adults (Geelhand et al., 2023). Posts written in English that used identity-first language also showed the highest averages in terms of metadata (4.15 replies, 6.58 reposts, 91.55 likes, and 3453.76 views). Sentiment across English posts remained largely neutral regardless of linguistic framing.

Posts in Spanish displayed the strongest medical framing, echoing findings by Montiel-Nava et al. (2025), who demonstrated that deficit-based metaphors still dominate Spanish-language coverage of autism. However, the temporal data show steady growth in neuroaffirmative expressions (e. g. “Proudly autistic”) since 2023, consistent with the global expansion of neurodiversity narratives (Harrison and Mosen, 2025; Schuck et al., 2022). In the Anglophone sample, medical and neuroaffirmative frames appeared in roughly equal proportions, reflecting the coexistence of clinical and identity-based repertoires described by Botha and Cage (2022). French discourse similarly combined medical and affirming registers, with affective ambivalence around suffering and inclusion, echoing Chamak's observation that France (Davidson and Orsini, 2013) remains torn between psychiatric and activist vocabularies. Georgian and Norwegian data, while smaller, revealed the importance of translation and localization: studies of autism communication in Georgia (Doghadze and Gagoshidze, 2025) indicate that local discourse remains predominantly medical and diagnostic, with neurodiversity-related terminology only beginning to appear in academic and public contexts. In Norway, we observed a similar coexistence between medical and inclusion-oriented framings, with terms like *nevrodiversitet* beginning to appear (Chahboun et al., 2022; Løkke et al., 2019), where clinical understandings continue to dominate despite growing international influence. This dominance also appears in public health communication: in a recent Norwegian study, autistic participants expressed frustration with the deficit-oriented focus of official health sources, which led them to seek more neuroaffirmative perspectives on social media (Skafle et al., 2024).

Temporal analysis suggests a rising trajectory in neuroaffirmative discourse in recent years, in line with the growing use of hashtags such as #ActuallyAutistic. Studies of Twitter discourse show that users employing *#ActuallyAutistic* increasingly frame identity-based narratives rather than purely medical ones (Chatterjee et al., 2023; Jaiswal and Washington, 2024) and that organizations and individuals in the autism community adjust their language over time (Akbulut and Bird, 2025). TikTok analyses likewise show that highly viewed autism videos emphasize self-description and acceptance over pathology (Gilmore et al., 2024). Moreover, a recent systematic review found an increasing preference for neuro-affirming vocabulary over deficit-based language across autism research publications (Bottini et al., 2024). The temporal distribution observed in our corpus aligns with these broader shifts toward self-advocacy and positive identification.

The sentiment analysis further supports this interpretation: content from identity-first communities (e.g., posts using #ActuallyAutistic) skews more positive than comparison groups, whereas discussions tied to therapeutic/medical framings (e.g., Applied Behavior Analysis debates) tend to be neutral-to-negative and draw higher engagement for negative posts (Jaiswal and Washington, 2024; Malkin et al., 2024). Conversely, corpus studies of British newspapers demonstrate that medical framings often pair autism with terms of struggle and burden (Hull et al., 2020). Thus, sentiment patterns in our corpus suggest how linguistic framing may reinforce or challenge emotional hierarchies.

Patterns of online engagement also differed by language and discourse type. In English, identity-first posts attracted the highest average likes and reposts, whereas in Spanish and French, neutral or medical posts (often linked to institutional accounts) garnered greater visibility. These trends align with recent reviews showing that autistic-produced content receives greater engagement in English networks, while institutional messages dominate in other languages; similarly, experiential content on TikTok outperforms clinical material (Gilmore et al., 2024; Jawed et al., 2023). Differences in interaction across our corpus therefore appear consistent with structural inequalities in digital participation: linguistic communities with stronger activist networks (e.g., English) amplify neuroaffirmative discourse more effectively than those where institutional communication still sets the tone.

Overall, these results suggest that language choice in autism discourse can be interpreted as operating simultaneously at symbolic and sociopolitical levels. Using *autistic person* rather than *person with autism* indexes allegiance to the neurodiversity paradigm, while persisting medical idioms reproduce the biomedical model of deficit. As Bottini et al. (2024) and Zajic and Gudknecht (2024) note, academic publishing continues to reproduce ableist formulations even as public discourse evolves. Bridging this gap requires explicit reflexivity: researchers and institutions should justify their language choices, acknowledge autistic preferences, and include autistic contributors in authorship and peer review. The increasing requirement by journals such as *Autism* to report community involvement (Tan et al., 2025) represents a positive step toward linguistic and epistemic accountability.

From the perspective of wellbeing, these dynamics highlight how discursive environments shape the interpretative frameworks through which autism is understood by both autistic individuals and the wider public. Multilingual social media spaces therefore act not only as arenas of debate but also as environments where meanings attached to autism—and the social conditions that affect wellbeing—are continuously negotiated.

At the same time, our multilingual evidence underscores that neurodiversity is not a homogeneous global movement but a constellation of culturally translated ideas. Comparative and anthropological work shows that neurodiversity is taken up and reworked across settings (Cascio, 2015, 2020), and scholars have argued that the paradigm operates as a form of biosociality/strategic essentialism whose meanings shift with local histories and institutions (Ellis, 2023). Clinical and policy uptake also varies across cultural context and moral economies (Schuck et al., 2022).

Finally, methodological caveats must be considered. Studies based on social-media corpora are affected by sampling bias, algorithmic filtering, and unequal platform access. These limitations are well documented in computational social-science literature (Jungherr et al., 2017). In addition, because data collection was conducted by different team members, each responsible for searches in a specific language, results may have been influenced by user-level algorithmic variation on the platform. This decision was made to ensure linguistic competence and accurate identification and interpretation of content in each language, rather than assigning all searches to a single researcher unfamiliar with some of the linguistic contexts analyzed. To mitigate potential algorithmic differences, all searches were conducted within the same predefined time window, using the same search interface (“advanced search”) and identical query parameters across languages. Nevertheless, algorithmic personalization on X cannot be fully controlled, and retrieved content may partly reflect user-specific algorithmic histories. Our stratified keyword approach mitigated some of these issues; however, cross-platform triangulation (X, TikTok, Reddit, local forums) would provide a more comprehensive view of autistic discourse across digital ecologies. Moreover, because keyword-based searches necessarily shape which lexical items and frames become visible, the distributions observed here should be interpreted as patterns emerging within the defined search space rather than as exhaustive representations of autism discourse on the platform. Also, because different communities and language use hashtags and keywords in carrying ways, we may have missed relevant content. The cross-linguistic comparisons should also be interpreted with caution, due to cultural differences and potential variations in the translated terms and sentiment lexicons. Future mixed-method designs that integrate linguistic analysis with interviews or ethnography could further clarify how individuals consciously adopt or resist specific language models (Jungherr, 2018).

## 5 Conclusions

The multilingual comparison presented in this study shows that online discourse reflects and contributes to the ongoing redefinition of autism across languages. Rather than evidencing a temporal evolution grounded in longitudinal sampling, the reduced centrality of medical framings alongside the increased visibility of neuroaffirmative and identity-first language illustrates cross-linguistic differences in how autism is discursively constructed on social media. Documenting and respecting these differences is essential for developing a truly inclusive, cross-cultural understanding of neurodiversity.

Looking ahead, future research should move beyond X/Twitter to capture the broader media ecosystems where autistic voices and discourses circulate. Different digital spaces offer unique environments in which language, algorithms, and community norms interact to shape what “autism” means in everyday life. Across these digital spaces, including AI systems, autism discourse is increasingly mediated. Incorporating these systems alongside public-health communication and autistic-led initiatives may clarify how neuroaffirmative perspectives circulate, challenge deficit narratives, and reshape public understandings of autism across cultures. Such work would not only expand the empirical base of autism discourse studies but also strengthen their ethical and social relevance.

## Data Availability

The datasets presented in this article are not readily available because the datasets generated and analyzed during the current study consist of publicly available posts from X/Twitter. Due to platform terms of service, the full dataset cannot be publicly shared, but it is available from the corresponding author on reasonable request Requests to access the datasets should be directed to Patricia López-Resa Patricia.lopezresa@uclm.es.
