# Supplementary material for: Discursive constructions of autism on social media: a multilingual analysis across five languages
Source: Front Psychol. 2026 Apr 17;17:1769965. doi: 10.3389/fpsyg.2026.1769965 (PMC13135461; doi:10.3389/fpsyg.2026.1769965)
Supplement: Supplementary file 1 [file Supplementary_file_1.docx]

Supplementary Material


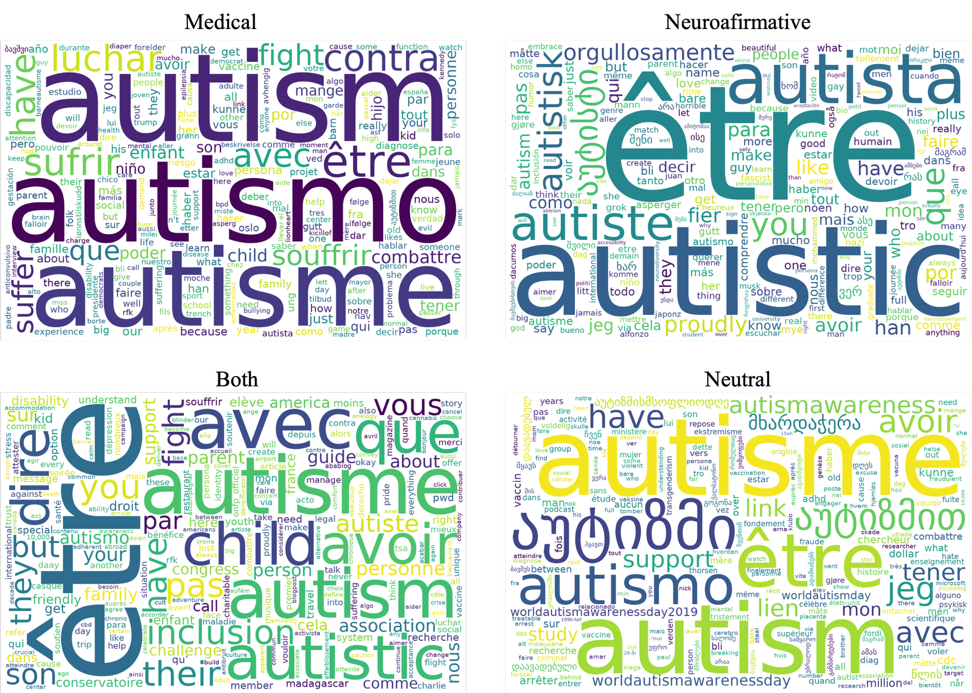


Supplementary Figure 1. Word clouds illustrating cross-linguistic lexical patterns grouped by theoretical linguistic model (medical, neuroaffirmative, both, and neutral). For each model, word size is proportional to the relative frequency of lexical items within the corresponding subset of posts, aggregated across the five languages analysed. Only the most frequent lexical items within each model are displayed.


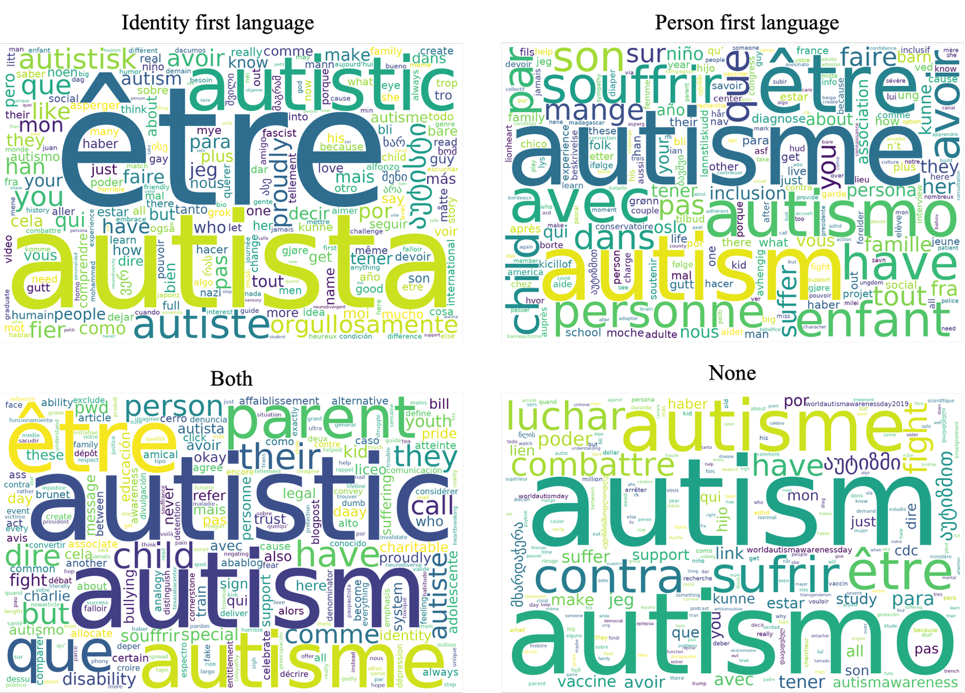


Supplementary Figure 2. Word clouds illustrating cross-linguistic lexical patterns grouped by linguistic style (identity-first, person-first, and neutral), highlighting relative lexical frequencies across languages. For each linguistic style, word size is proportional to the relative frequency of lexical items within the corresponding subset of posts, aggregated across the five languages analysed. Only the most frequent lexical items within each linguistic style are displayed.


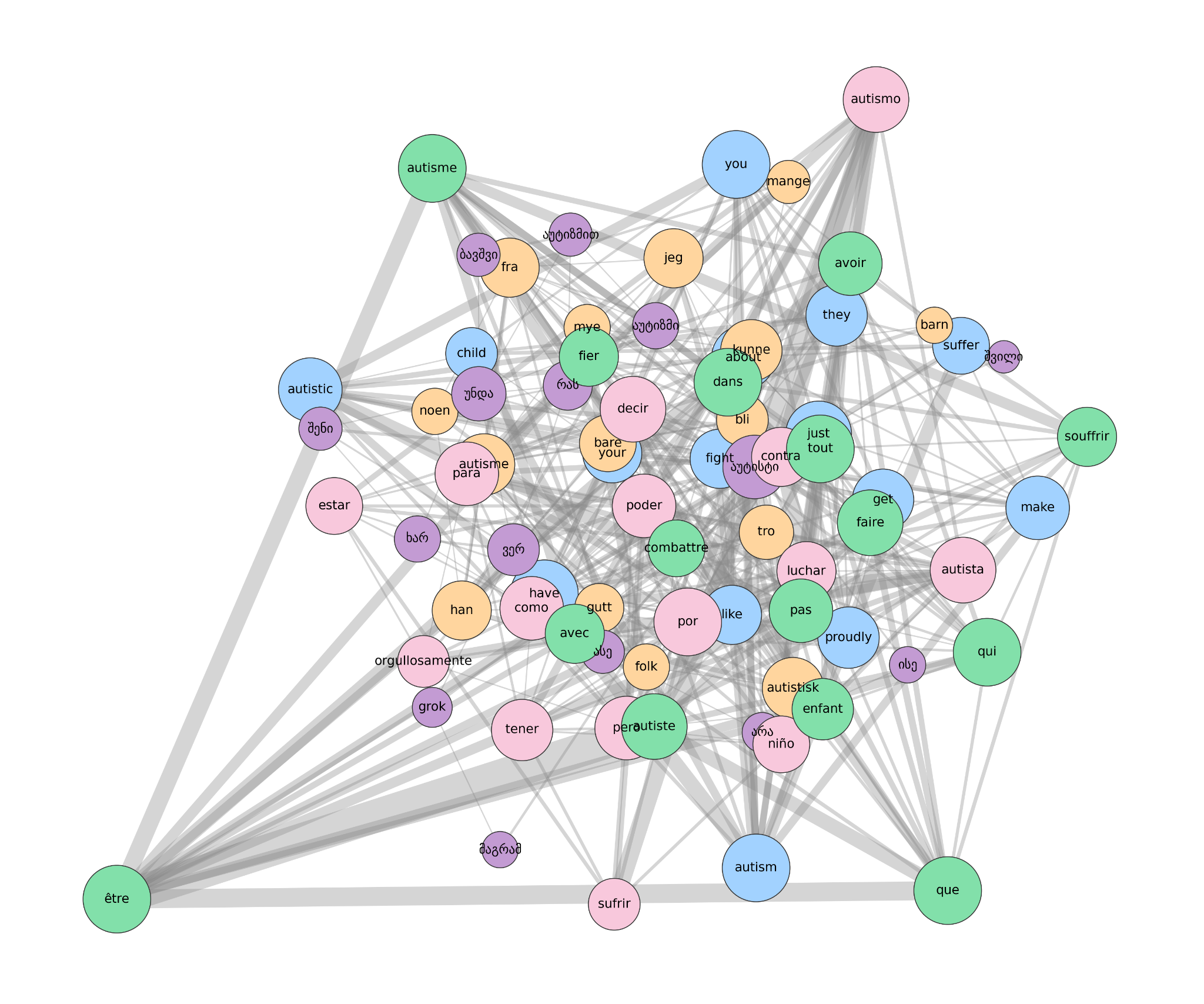


**Supplementary Figure 3.** Network illustrating lexical and conceptual associations across discursive frameworks (medical, neuroaffirmative, and neutral). Nodes represent lexical items and edges indicate co-occurrence within posts. Node colour denotes language (French = green, English = blue, Norwegian = orange, Spanish = pink, Georgian = purple), while edge thickness reflects the relative strength of co-occurrence between terms. The network highlights cross-linguistic clustering and shared lexical associations across discursive frameworks


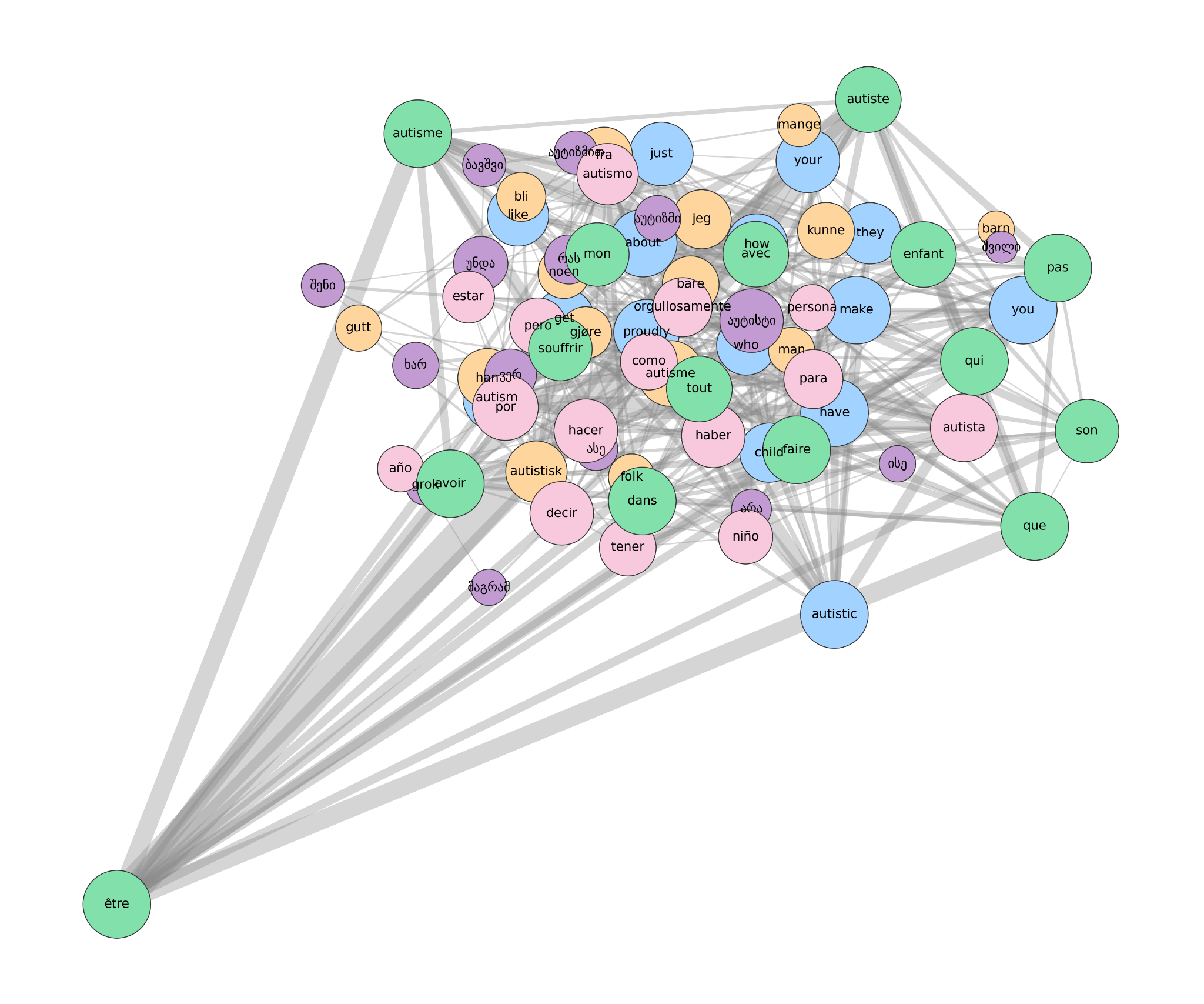


**Supplementary Figure 4.** Network illustrating lexical and conceptual associations across linguistic styles (identity-first, person-first, and neutral). Nodes represent lexical items and edges indicate co-occurrence within posts. Node colour denotes language (French = green, English = blue, Norwegian = orange, Spanish = pink, Georgian = purple), while edge thickness reflects the relative strength of co-occurrence between terms. The network visualises how lexical items cluster across linguistic styles, highlighting shared and style-specific associations across languages.
